# Nausea-Induced 5-HT Release in the Interoceptive Insular Cortex and Regulation by Monoacylglycerol Lipase (MAGL) Inhibition and Cannabidiol

**DOI:** 10.1523/ENEURO.0256-18.2018

**Published:** 2018-07-31

**Authors:** Cheryl L. Limebeer, Erin M. Rock, Keith A. Sharkey, Linda A. Parker

**Affiliations:** 1 Department of Psychology and Neuroscience Program, University of Guelph, Guelph, Ontario N1G 2W1, Canada; 2 Hotchkiss Brain Institute, Department of Physiology and Pharmacology, University of Calgary, Calgary, Alberta T2N 4N1, Canada

**Keywords:** cannabinoid, interoceptive insular cortex, microdialysis, nausea, rat, serotonin

## Abstract

Using the rat conditioned gaping model of nausea, the interoceptive insular cortex (IIC) has been identified as a critical site for the regulation of lithium chloride (LiCl)-induced nausea. Indirect evidence supports a model where serotonin (5-HT) acts on postsynaptic 5-HT_3_ receptors and its release is suppressed by elevating 2-arachidonylglycerol (2-AG) by monoacylglycerol lipase (MAGL) inhibition to suppress nausea. Here, we directly test the hypothesis that systemic LiCl elevates 5-HT in the IIC, and this is prevented by pretreatments that reduce 5-HT release. Using male Sprague Dawley rats, LiCl (but not saline), elevated 5-HT selectively in the IIC, for 20 min after LiCl administration (127.2 mg/kg, i.p.). Systemic pretreatment with the MAGL inhibitor, MJN110, prevented the LiCl-induced elevation of 5-HT in the IIC. Systemic cannabidiol (CBD), which reduces LiCl-induced nausea by acting at 5-HT_1A_ somatodendritic autoreceptors, also prevented LiCl-induced elevation of 5-HT in the IIC. Since 5-HT_3_ receptor agonists delivered to the IIC produce nausea, we tested and confirmed the hypothesis that the intra-IIC administration of 5-HT_3_ receptor antagonist, ondansetron, but not MJN110, would prevent LiCl-induced conditioned gaping reactions produced by intra-IIC administration of the 5-HT_3_ receptor agonist, *m-*chlorophenylbiguanide (mCPBG). Finally, we demonstrate that exposure to a LiCl-paired flavor (but not a saline-paired flavor) produces elevated 5-HT release in the IIC, while rats display conditioned gaping reactions. These results confirm that LiCl-induced nausea is triggered by elevated 5-HT release in the IIC and is attenuated by treatments that reduce 5-HT availability in this region.

## Significance Statement

Understanding of the neurobiology of nausea has lagged behind that of the neurobiology of vomiting. Here we demonstrate for the first time that a nauseating drug produces an elevation of serotonin in the brain region that mediates nausea in humans, the interoceptive insular cortex (IIC). This elevated serotonin (and nausea) is prevented by pretreatment with a drug that elevates the endocannabinoid (eCB), 2-arachidonylglycerol (2-AG), in this region. As well, pretreatment with the non-psychoactive cannabinoid, cannabidiol, acts to reduce forebrain release of serotonin (and nausea) triggered by the nauseating drug. These results strongly suggest serotonin serves as a trigger to produce the sensation of nausea in the IIC and cannabinoids act to prevent this trigger to regulate nausea.

## Introduction

While current anti-emetic therapies are highly effective in reducing vomiting, they are less effective in treating chemotherapy-induced nausea (Hickok et al., 2003; Foubert and Vaessen, 2005; Ballatori et al., 2007), because of a poor understanding of the neurobiology of nausea. We have demonstrated that conditioned gaping elicited by a nausea-paired taste in the taste reactivity test in the rat (initially identified by Grill and Norgren, 1978) is a highly selective model of nausea and have used it to explore the neural circuits involved in the regulation of nausea, including serotonergic (5-HT) and endocannabinoid (eCB) mechanisms (Parker, 2014).

The insular cortex (IC) is a critical region for generating the sensations of nausea (Penfield and Faulk, 1955; Kiefer and Orr, 1992; Contreras et al., 2007; Napadow et al., 2013; Sclocco et al., 2016) and disgust (Calder et al., 2000, 2001). Electrophysiological and anatomic studies in rats have determined that the IC is the cortical site of topographical input of visceral (posterior granular or interoceptive IC; IIC) and gustatory input (anterior dysgranular or gustatory IC; GIC) and their convergence (agranular IC; Cechetto and Saper, 1987; Hamilton and Norgren, 1984; Kosar et al., 1986; Allen et al., 1991). Growing evidence suggests that elevated 5-HT in the IIC triggers nausea and treatments that reduce 5-HT in the IIC suppress nausea. In a double dissociation study, we found that intracranial administration of the 5-HT_3_ receptor antagonist, ondansetron, into the IIC attenuated lithium chloride (LiCl)-induced conditioned gaping, but not taste avoidance (Tuerke et al., 2012a). Conversely, ondansetron delivered into the GIC attenuated LiCl-induced taste avoidance, but not conditioned gaping. Direct delivery of a 5-HT_3_ receptor agonist into these regions produced the opposite effect and produced nausea on its own in the IIC. These data provide strong evidence that serotonergic input to the IIC is necessary for the production of nausea-induced conditioned gaping. Here, we evaluate the hypothesis that systemic LiCl elevates 5-HT in the IIC and this is prevented by pretreatments that reduce 5-HT release by directly measuring 5-HT release in the IIC during episodes of LiCl-induced nausea.

The anti-nausea effects of cannabinoids (Sticht et al., 2016) and treatments that reduce the release of 5-HT, such as cannabidiol (CBD), cannabidiolic acid (CBDA), and 8-hydroxy-2-(di-npropylamino) tetralin (8-OH-DPAT; Limebeer and Parker, 2003; Rock et al., 2012; Bolognini et al., 2013), may be mediated by their action in the IIC. There is considerable evidence that CB_1_ receptors are localized on presynaptic terminal endings of 5-HT releasing neurons (Häring et al., 2007, 2013); therefore, it is possible that activation of CB_1_ receptors in the IIC suppresses the release of nausea-inducing 5-HT. The potent cannabinoid agonist, HU-210, delivered into the IIC (but not the GIC), reduced LiCl-induced conditioned gaping reactions by a CB_1_ receptor mechanism (Limebeer et al., 2012). More recently, we found that selective elevation of the eCB, 2-arachidonylglycerol (2-AG), but not anandamide (AEA), in the IIC, by pretreatment with the monoacylglycerol lipase (MAGL) inhibitor, MJN110, reduced LiCl-induced conditioned gaping, by a CB_1_ receptor mechanism of action (Sticht et al., 2016). As well, 2-AG (but not AEA) is elevated and c-fos is activated in the IIC during an acute episode of LiCl-induced nausea. CBD, CBDA, and 8-OH-DPAT all reduce nausea-induced conditioned gaping by acting at somatodendritic 5-HT_1A_ autoreceptors in the dorsal raphe nucleus (Rock et al., 2012); this action results in reduced firing of 5-HT afferent neurons and a corresponding decrease in the release of 5-HT in terminal regions (Verge et al., 1985; Blier and de Montigny, 1987). Therefore, we predict that MJN110 and CBD will reduce the elevation in 5-HT in the IIC which triggers LiCl-induced nausea. Although MJN110 is predicted to reduce LiCl-induced release of 5-HT, it would not be expected to interfere with the nausea produced by an agonist of postsynaptic 5-HT_3_ receptors delivered to the IIC. Finally, we evaluate whether exposure to a LiCl-paired flavor will conditionally elevate 5-HT in the IIC.

## Materials and Methods

### Subjects

A total of 147 male Sprague Dawley rats were obtained from Charles River Canada and pair-housed in polycarbonate cages (44 × 25 × 21 cm), with Bed-0’Cobs bedding on the floor of the cage. Subjects were provided with food pellets (Highland Rat Chow) and water ad libitum. The animal quarters were kept on a reversed 12/12 h light/dark cycle (lights on from 7 P.M. to 7 A.M.) and maintained at 22 ± 2°C and 45 ± 20% relative humidity. All animals were handled before testing and the guidelines set out by the Canadian Council on Animal Care Committee and the Animals for Research Act were followed. The experiments were approved by the University of Guelph Animal Care Committee.

### Drugs

Drugs were administered systemically, by intraperitoneal injection, or intracranially into the IIC. LiCl was used as the nausea-inducing agent, at a dose of 127.2 mg/kg (20 ml/kg of 0.15 M solution), the optimal dose for inducing conditioned gaping (Zalaquett and Parker, 1989). LiCl, the standard nausea-inducing drug (Garcia et al., 1974), is a relatively non-toxic compound producing malaise for ∼45 min and can be chronically administered (Tuerke et al., 2012b). Control groups for LiCl were injected with an equal volume saline as vehicle (VEH) controls. MJN110 and CBD were prepared in a VEH consisting of a 1:1:18 mixture of ethanol:Tween 80:saline. MJN110 or CBD were first dissolved in ethanol in a graduated cylinder, then Tween 80 is added to the solution and the ethanol is evaporated off with a nitrogen stream after the saline is added. The final VEH consisted of 1:9 (Tween:saline) which was used as the VEH control for the MJN110 and the CBD pretreatments. For systemic administration, the dose of MJN110 was 10 mg/kg (Parker et al., 2015; Sticht et al., 2016) administered at a volume of 1 ml/kg, for intracranial administration the dose of MJN110 was 2μg/μl and microinfused into the IIC at 0.5 μl/min for 2 min (Sticht et al., 2016). The dose of CBD was 5 mg/kg administered at a volume of 1 ml/kg (Rock et al., 2012). The 5-HT_3_ agonist, *m*-chlorophenylbiguanide (mCPBG) and the 5-HT_3_ antagonist, ondansetron, were prepared for intracranial administration in sterile saline solution at concentrations of 30 and 0.1 μg/μl, respectively (Tuerke et al., 2012a). They were both infused at a rate of 0.5 μl/min for 2 min.

### Surgical procedures

In experiments 1 and 3, the rats were implanted with a unilateral and in experiment 2 with bilateral indwelling guide cannulae into the IIC (region of interest), or into the GIC (control region), while under isoflurane anesthesia for later insertion of a microdialysis probe. The stereotaxic surgery procedure has been previously described (Tuerke et al., 2012a). The guide cannula (21 gauge, 6 mm below pedestal) was set at a divergent 10° angle and lowered into the IIC using the following coordinates from bregma: -0.5 mm AP, 5.0 mm ML, -4.5 mm DV (Contreras et al., 2007); half of the rats received cannulae in the left hemisphere and half in the right hemisphere. A control experiment evaluated microdialysis samples taken from the GIC (from bregma: +2.5 mm AP, 5.0 mm ML, -4.5 mm DV), which we have shown is not involved in nausea-induced gaping (Limebeer et al., 2012; Tuerke et al., 2012a). In experiments 2 and 3, on the day of intracranial surgery, rats were also implanted with an intraoral cannula for the oral delivery of the saccharin solution in the taste reactivity conditioning/testing trials according to the procedure previously described by Limebeer et al. (2012).

### Histology

**Figure 1.**
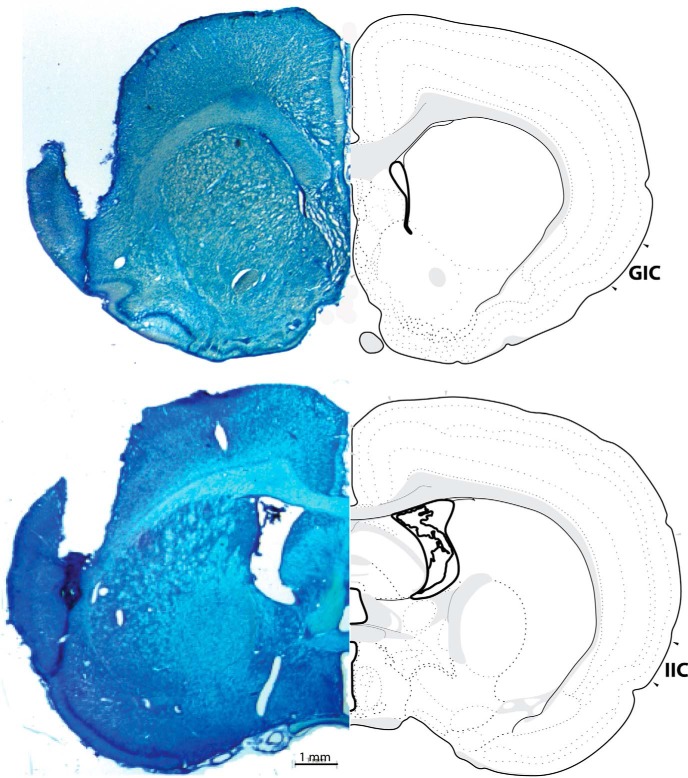
Representative photomicrograph of probe placement into the GIC and the IIC. Accurate GIC (control region) placements were between 1.68 and 1.20 mm anterior to bregma. Accurate IIC placements were between -0.24 and -0.72 mm posterior of bregma.

Guide cannulae placements were evaluated by histology. Rats were deeply anaesthetized using an 85 mg/kg injection of Euthansol (Intervet Canada Corp.) followed by transcardial perfusion with phosphate buffered saline (0.1 M) and 4% formalin. The brains were removed and stored at 4°C in 4% formalin solution for 24–48 h after which they were placed in a 20% sucrose solution overnight at room temperature. The brains were then sliced in 60-µm sections using a CM1850 Leica cryostat and relevant sections were mounted on glass microscope slides. The tissue was stained with cresyl violet 24 h later and examined for accurate cannula placement using a Leica MZ6 Stereomicroscope with a Leica DFC420 Digital Camera and Leica Application Suite software. Rats with improper cannula placements, such as those located outside of the target region were excluded from the behavioural analyses. All reported group n’s refer to the rats with verified cannulae placements. Accurate IIC placements were between -0.24 and -0.72 mm posterior of bregma. A representative photomicrograph of an IIC (the region of interest) placed probe is presented in Figure 1. Accurate GIC placements (control region) were between 1.68 and 1.20 mm anterior to bregma. A total of 21 rats had misplaced cannulae in the reported experiments; therefore the total number of rats included in the analyses was 126.

### In vivo microdialysis and HPLC detection of 5-HT

The microdialysis probes consist of 2.5 mm in length of semipermeable dialysis membrane from Spectra/Por *in vivo* Microdialysis Hollow Fibers, 2 μm OD, 13,000 MW cutoff. On the day of sampling, a microdialysis probe was inserted into the guide cannula directed to the IIC or the GIC. Artificial CSF (aCSF; 147 mM NaCl, 2.8 mM KCl, 1.2 mM CaCl_2_, and 1.2 mM MgCl_2_; pH 7.4) was perfused at a rate of 0.6 μl/min for a period of 300 min, after an acclimatization of 120 min, samples were collected and frozen on dry ice every 20 min for a total of 300 min.

The dialysate from the samples were analyzed for 5-HT using the Eicom HTEC-510 HPLC/ECD system (Eicom USA). Each sample was extracted from the vial and loaded on a C-18 reverse-phase column (PP-ODS II, 4.6 × 30 mm, Eicom USA) using a manual injection port (Rheodyne 9725i; 20-µl loop). The column was maintained at a temperature of 25°C with a mobile phase [0.1 M phosphate buffer pH 5.4, including 1.5% methanol, 500 mg/l decansulfonate sodium salt (DSS), and 50 mg/l 2Na-EDTA] set at a flow rate of 0.6 μl/min. Electrochemical detection of 5-HT was determined using a graphite working electrode (WE-3G, Eicom USA) maintained at a potential of +450 mV relative to an Ag/AgCl reference electrode (RE-500, Eicom USA).

### Behavioral procedures

#### Experiment 1: effect of LiCl on 5-HT release in the IIC or GIC (control area) and effect of pretreatments with MJN110 or CBD

On the day of sampling, rats were placed into the opaque black Plexiglas microdialysis chambers (60 × 40 × 40 cm) with Bed-0’Cobs bedding on the plastic floors. A microdialysis probe was inserted into the guide cannula directed to the IIC or the GIC and aCSF was perfused at a rate of 0.6 µl/min. After an acclimatization of 120 min, baseline samples were collected and frozen every 20 min for 60 min. These three measures served as the baseline measures to be used in the analyses. To compare the subsequent effects of pretreatment with MJN110 and CBD in subsequent experiments, the rats were then injected with the VEH (1:9 Tween 80:saline) 1 h before receiving either LiCl (*n* = 10 in IIC, *n* = 7 in GIC) or saline (*n* = 10 in IIC, *n* = 7 in GIC). Samples continued to be collected every 20 min for 240 min after the pretreatment (VEH) injection.

Once it was determined that LiCl elevated 5-HT in the IIC, the effect of pretreatment with MJN110 or CBD 1 h before receiving either LiCl or saline on the elevation of 5-HT in the IIC produced by LiCl was determined. The following groups were evaluated: MJN saline (*n* = 11), MJN LiCl (*n* = 11), CBD saline (10), CBD LiCl (9), and compared with the previously collected groups VEH-saline (*n* = 11) and VEH-LiCl (*n* = 9).

#### Experiment 2: effect of MJN110 or ondansetron in the IIC on conditioned gaping elicited by IIC infusion of 5-HT_3_ receptor agonist mCPBG

Tuerke et al. (2012a) demonstrated that activation of the postsynaptic 5-HT_3_ neurons in the IIC during saccharin-LiCl conditioning is critical for the development of conditioned gaping reactions. If MAGL inhibition-induced elevated 2-AG acts to reduce nausea by reducing LiCl-induced release of 5-HT in the IIC, then MJN110 should not prevent gaping produced by an IIC infusion of the 5-HT_3_ receptor agonist mCPBG. On the other hand, ondansetron administration in the IIC should prevent mCPBG-induced conditioned gaping, because it blocks postsynaptic 5-HT_3_ receptors. To test this hypothesis, during each of two conditioning trials, rats were infused in the IIC with VEH, MJN110 (2 µg/µl), or OND (0.1 µg/µl) bilaterally and 1 h later, they received a pairing of saccharin with IIC infusion of mCPBG (30 µg/µl), which produces conditioned gaping (Tuerke et al., 2012a).

Following recovery from surgery, rats were placed in the taste reactivity apparatus (see Tuerke et al., 2012a for procedural details) with their intraoral cannuale attached to an infusion pump to allow intraoral delivery of fluid across their tongue. Following 3 min in the taste reactivity chamber, the rats were infused with water at the rate of 1 ml/min for 3 min. Twenty-four hours after the adaptation trial, the rats received two conditioning trials spaced 72 h apart followed by a drug free test trial 72 h later. These trials were identical to the adaptation trial, except that the rats were intraorally infused with 0.1% saccharin solution instead of water and their orofacial and somatic responses were recorded from a mirror beneath the chamber. An observer who was blind to the experimental condition of the rat scored the orofacial and somatic responses using *The Observer* (Noldus Information Technology Inc.). The groups were randomly assigned to VEH-saline (*n* = 10), VEH-mCPBG (*n* = 10), MJN110–mCPBG (*n* = 8), OND-mCPBG (*n* = 10). MJN110 pretreatment was administered 60 min before each conditioning trial, whereas all other pretreatments were administered immediately before conditioning trials 1 and 2 (Tuerke et al., 2012a). Immediately following the saccharin infusion, each rat was microinfused with mCPBG or saline into the IIC. Following all microinfusions, the injector which extends 2 mm below the tip of the guide cannula, was left in place for 1 min. Following the test trial, the rats were perfused and the brains removed for histologic examination. An observer who was blind to the experimental condition of the rat scored the recorded tapes for the frequency of gaping reactions (wide open triangular shaped mouth revealing incisors) using *The Observer* (Noldus Information Technology Inc.).

#### Experiment 3: effect of exposure to a LiCl-paired saccharin solution on 5-HT release in the IIC

Experiment 3 evaluated the potential of exposure to a LiCl-paired flavor to elicit elevated 5-HT in the IIC, when they were gaping in the microdialysis chambers. To ensure that the rats would maintain gaping reactions to the LiCl-paired saccharin solution across the 20-min test, they were given three conditioning trials before testing. To prevent the establishment of an association between the taste reactivity chambers and LiCl-induced nausea, the rats received a total of four daily adaptation trials to the chambers during which their intraoral cannulae were attached to the infusion pump (Model KDS100, KD Scientific) and they were infused with water at the rate of 1 ml/min for 2 min. Following the adaptation trial, the rats were conditioned every 72 h. On each conditioning trial, the rat was placed in the taste reactivity chamber for 1 min and then was infused with 0.1% saccharin solution (1 ml/min) for 2 min followed immediately by an intraperitoneal injection of LiCl (*n* = 7) or saline (*n* = 6). To ensure equal exposure to the experience of illness, rats received non-contingent home cage injections (24 h after conditioning trial 1 and 24 h before each of conditioning trials 2 and 3) such that the LiCl-conditioned rats received saline and the saline-conditioned rats received LiCl.

Ninety-six hours after the final taste reactivity conditioning trial, the rats were given the drug-free taste reactivity test trial in the microdialysis chambers with a clear glass floor in place of the plastic floor with bedding. Before placement in the chambers, microdialysis probes were inserted into the guide cannula directed to the IIC and aCSF was infused at the rate of 0.6 µl/min. After an acclimatization of 120 min, baseline samples were collected and frozen every 20 min for 120 min. These six measures served as the baseline measures to be used in the analyses. Then the intraoral cannula was attached to the infusion pump and the rat was intraorally infused with saccharin solution for 20 min at the rate of 0.5 ml/min. Its orofacial and somatic reactions were recorded during the test trial from a mirror at a 45° angle beneath the microdialysis chamber. The tapes were later scored for gaping reactions (wide open triangular mouth with bottom incisors exposed). Additionally, seconds of active locomotion (forward movement in the chamber) and rearing (both paws off the floor of the cage) were summed as a total activity measure.

### Experimental design and statistics

The concentration of 5-HT in the dialysate samples was converted to percentage baseline (determined by the mean pg/μl of the last three baseline readings before injection 1 in experiments 1 and 2 and the mean pg/μl of the six baseline readings before saccharin infusion in experiment 3) entered into mixed factor ANOVAs with the within group factor of time of sample (a total of 15). First, the effect of LiCl on elevation of 5-HT in the IIC or the GIC was separately analyzed as a 2 (treatment: LiCl or saline treatment) × 15 (time measures) factors ANOVA. Then the this baseline response was compared with groups that received pretreatments in a 3 (pretreatment) × 2 (treatment) × 15 (time measures) mixed factors ANOVA. In experiment 2, the number of gapes displayed by each rat during both conditioning trials and the test trial was entered into a 4 (group: VEH-VEH, VEH-mCPBG, OND-mCPBG, MJN-mCPBG) × 3 (trial: C1, C2, test) mixed factor ANOVA. Finally, to determine whether LiCl-paired saccharin would conditionally elevate 5-HT in the IIC, the percentage baseline 5-HT measures were entered into a 2 (conditioning group: Sac->LiCl or Sac->saline) × 15 (time) mixed factors ANOVA. As well the number of gapes and number of seconds displaying total activity (active locomotion and rearing combined) were analyzed by independent *t* tests. *Post hoc* Tukey HSD tests were used when appropriate.

## Results

### Experiment 1: effect of LiCl on 5-HT release in the IIC or GIC and effect of pretreatments with MJN110 or CBD

**Figure 2.**
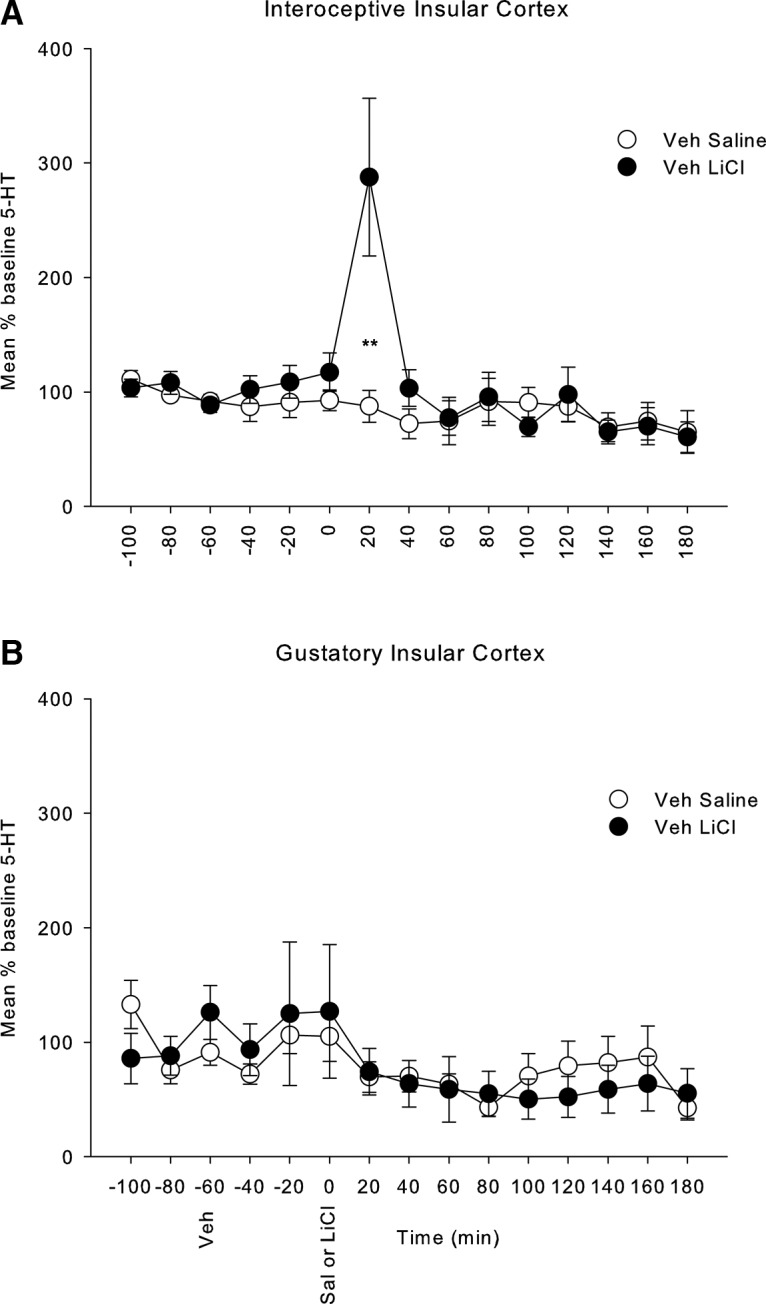
Mean (±SEM) % baseline 5-HT in rats with microdialysis probes placed in the (***A***) IIC and in the (***B***) GIC. Following 120 min of acclimatization, samples were collected every 20 min for a total of 300 min; the first three samples served as the baseline samples which were used to convert each score into a % baseline measure. The rats were injected intraperitoneally with a VEH immediately following the final baseline measure and with equivolume LiCl (127.2 mg/kg, i.p.) or saline 60 min later. Stars indicate a significant difference between LiCl and saline, **p < 0.025.

LiCl treatment elevated 5-HT in the IIC, but not the GIC, for the first 20-min period of dialysate collection. Figure 2 presents the mean (±SEM) percentage of baseline 5-HT during each 20-min collection period in the IIC (1a) and in the GIC (1b). The 2 × 15 mixed factors ANOVA for the percentage of baseline 5-HT in the IIC revealed a significant main effect of time, *F*_(14,252)_ = 5.2; *p* < 0.001 and a significant group by time interaction, *F*_(14,252)_ = 4.1; *p* < 0.001. Subsequent comparison tests revealed that the only interval that the groups differed was during the first 20 min after injection (*p* < 0.025). On the other hand, the 2 × 15 mixed factors ANOVA for the percentage of baseline 5-HT in the GIC revealed only a significant main effect of time, *F*_(14,168)_ = 2.7; *p* < 0.01, but no effects of group.

**Figure 3.**
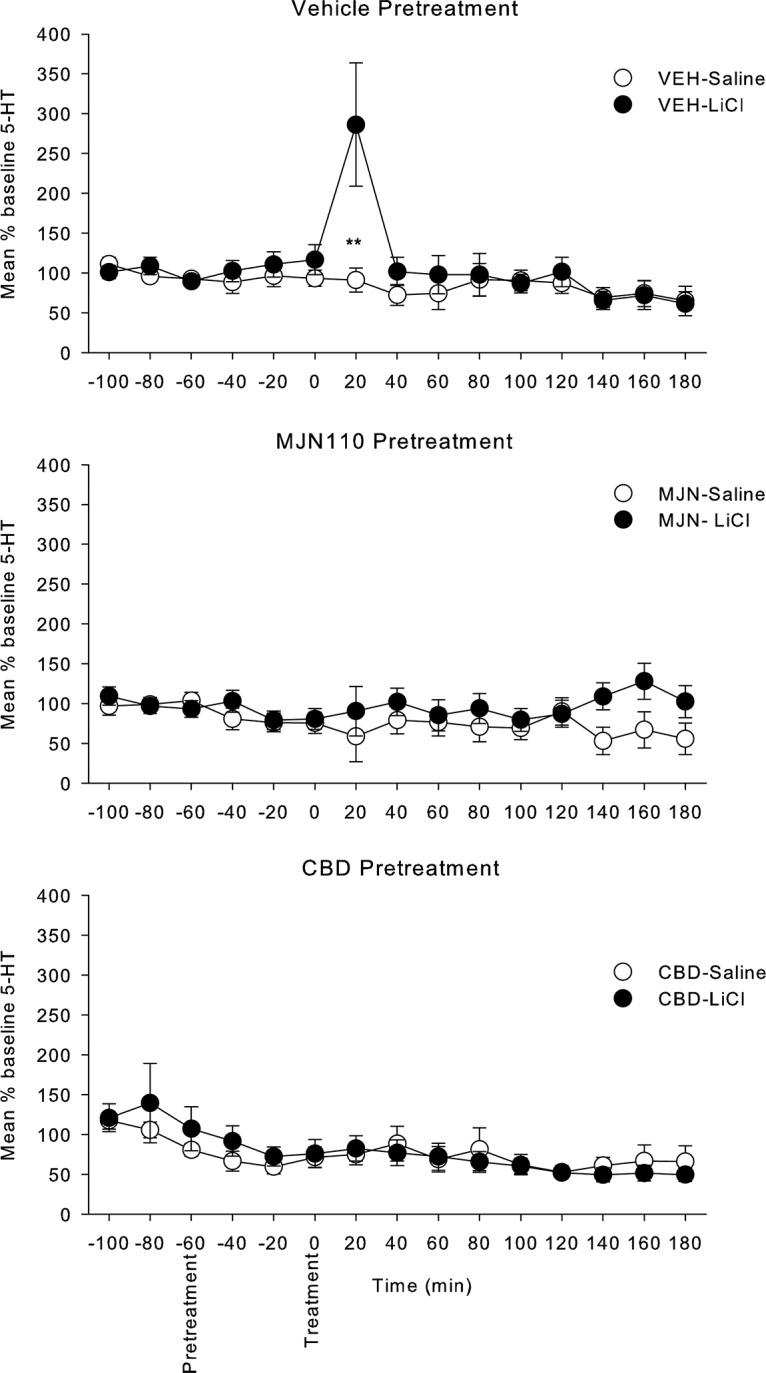
Mean (±SEM) % baseline 5-HT in rats with microdialysis probes placed in the IIC. Following the initial three baseline measures, the rats were injected intraperitoneally with VEH, MJN110 (10 mg/kg), or CBD (5 mg/kg) followed 60 min later with LiCl or saline. Stars indicate a significant difference between LiCl and saline, **p < 0.025.

Both MJN110 and CBD pretreatment reduced the release of 5-HT during the first 20 min after LiCl. Figure 3 presents the mean (±SEM) percentage of baseline 5-HT during each 20-min collection period for the VEH, MJN110, and CBD pretreatment groups. The mixed factors ANOVA revealed a significant main effect of time, *F*_(14,770)_ = 5.4; *p* < 0.001, pretreatment × time interaction, *F*_(28,770)_ = 3.1; *p* < 0.001, treatment × time interaction, *F*_(14,770)_ = 2.2; *p* < 0.01, and pretreatment × treatment × time interacton, *F*_(28,770)_ = 2.4; *p* < 0.001. The three-way interaction was subsequently evaluated by conducting separate 2 × 15 ANOVAs for the treatment condition for each pretreatment condition separately (as depicted in Fig. 3). For the VEH pretreatment group, there was a significant main effect of time, *F*_(14,252)_ = 5.2; *p* < 0.001, and a treatment by time interaction, *F*_(14,252)_ = 4.1; *p* < 0.001, with group LiCl displaying higher levels than group saline only during the first 20 min following LiCl treatment (*p* < 0.025). For the MJN110 pretreatment group, the 2 × 15 ANOVA revealed no significant effects, with LiCl and saline groups not differing at any interval of testing. For the CBD pretreatment group, the mixed factor ANOVA only revealed a significant main effect of time, *F*_(14,238)_, with 5-HT levels decreasing across the test overall, but this factor did not interact with the treatment condition.

### *Experiment 2*: *effect of MJN110 or ondansetron in the IIC on conditioned gaping elicited by IIC infusion of 5-HT_3_ receptor agonist mCPBG*

**Figure 4.**
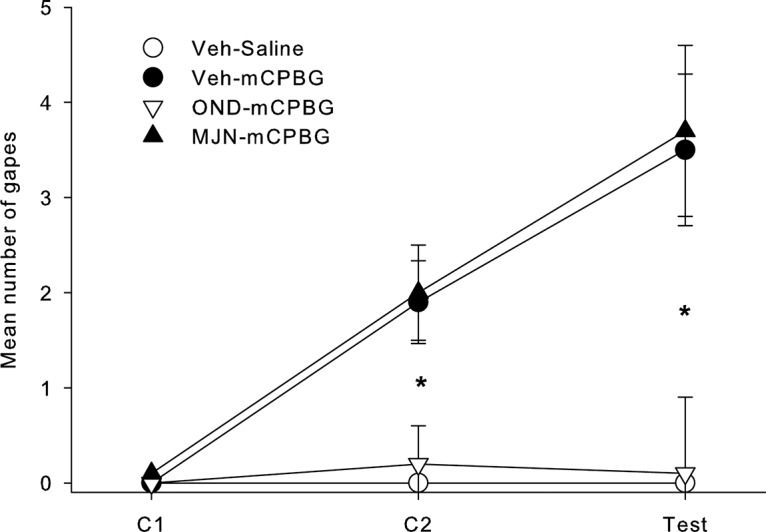
Mean (±SEM) number of gapes displayed by rats in the various groups in experiment 2. Stars indicate a significant difference at *p < 0.05.

The mean number of gapes displayed between the groups across all trials can be seen in Figure 4. Pre-treatment with the 5-HT_3_ receptor antagonist OND, but not with MJN110, prevented a conditioned gaping response that is produced by the 5-HT_3_ receptor agonist mCPBG. The 3 × 4 mixed factor ANOVA with the between groups factor of group and within groups factor of trial revealed a significant main effect of group, *F*_(3,34)_ = 9.4; *p* < 0.001, trial *F*_(2,68)_ = 13.0; *p* = 0.001=, and a group by trial interaction *F*_(6,68)_ = 4.1; *p* < 0.001. Single factor ANOVAs revealed a significant main effect of group in the second conditioning trial *F*_(3,34)_ *=* 6.3; *p* = 0.002, and test trial *F*_(3,34)_ = 6.3; *p* = 0.002; subsequent Tukey HSD tests revealed that on both C2 and the test trial, groups VEH-mCBG and MJN-mCPBG gaped significantly more than either group VEH-saline or OND-mCPBG (*p*s < 0.05).

### Experiment 3: conditioned release of 5-HT in IIC

**Figure 5.**
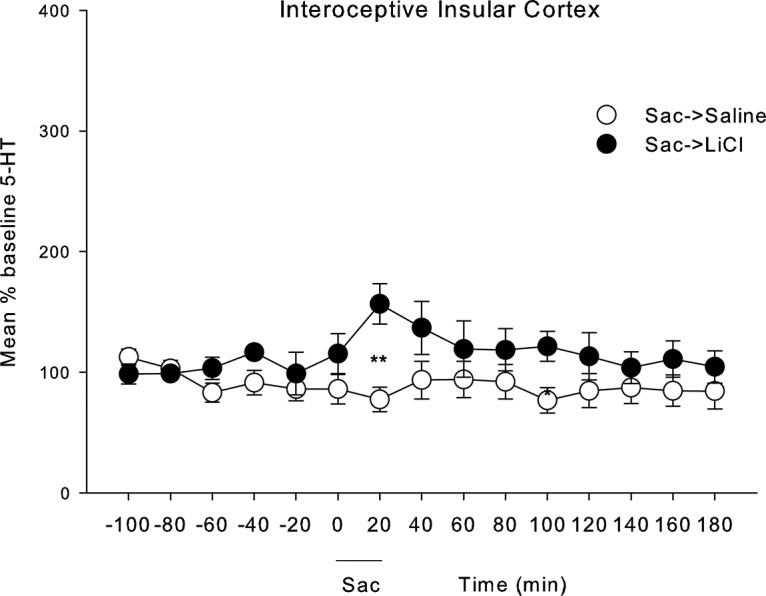
Mean (±SEM) % baseline 5-HT in rats with microdialysis probes placed in the IIC over the 300 min of sample collection every 20 min. The first six measures served as baseline measures which were used to convert each score into a % baseline measure. Following the final baseline measure, the rats were intraorally infused with saccharin for 20 min, and samples continued to be collected for the remainder of the 300-min session. Stars indicate a significant difference at ***p < 0.01.

Rats exposed to LiCl-paired saccharin displayed higher levels of 5-HT release during the saccharin exposure period than rats exposed to saline-paired saccharin. Figure 5 presents the mean (±SEM) percentage of baseline 5-HT during each sampling interval among the rats exposed to saccharin previously paired with LiCl or saline. The 2 × 15 mixed factors ANOVA revealed only a significant group × time interaction, *F*_(14,154)_ = 2.04; *p* = 0.018. Subsequent *t* tests revealed that group Sac->LiCl showed elevated 5-HT relative to group Sac->saline during the 20 min taste reactivity test trial (*p* < 0.01), only. Table 1 presents the mean (±SEM) number of gapes and sec of general activity (active locomotion + rearing) displayed during the 20 min sampling period in the microdialysis chambers. As is apparent, the LiCl conditioned rats gaped more to the saccharin infusion than the saline conditioned rats (which did not gape), *t*_(11)_ = 5.705; *p* < 0.001. The groups did not differ in overall general activity level, *t*_(11)_ = 0.598; *p* > 0.05.

**Table 1.** Mean (±SEM) number of gapes and seconds of activity displayed by the LiCl and saline conditioned groups during the 20-min infusion of saccharin solution in the microdialysis chamber in experiment 3

	Gapes	Activity (s)
Saline	0.0 (+0.00)	130.83 (+33.46)
LiCl	45.43 (+7.33)^***^	158.00 (+36.38)

*** *p* < 0.01.

## Discussion

LiCl, but not equivolume injections of saline, elicited an elevation of 5-HT in the IIC, but not in the adjacent GIC. This elevated 5-HT was prevented by pretreatment with the MAGL inhibitor, MJN110, or with the 5-HT_1A_ receptor agonist, CBD. Since intracranial administration of MJN110 into the IIC prevents LiCl-induced conditioned gaping reactions (Sticht et al., 2016), these results suggest that the suppression of nausea by MJN110 is mediated by its reduction of LiCl-induced release of 5-HT in this region. These results are consistent with reports that selective 5-HT lesions (with 5,7-DHT) in the IIC, but not the GIC, prevent LiCl-induced conditioned gaping reactions (Tuerke et al., 2012a). As well, administration of the CB_1_ receptor agonist, HU-210, into the IIC, but not the GIC, reduces LiCl-induced gaping reactions (Limebeer et al., 2012). Both the central effects of MJN110 and HU-210 are CB_1_ receptor mediated, because their anti-nausea effect is prevented by pretreatment with the CB_1_ receptor antagonist, AM251 (Limebeer et al., 2012; Sticht et al., 2016). CBD acts as a somatodendritic 5-HT_1A_ autoreceptor agonist in the dorsal raphe nucleus to reduce LiCl-induced conditioned gaping in rats (Rock et al., 2012). Within this region, 5-HT_1A_ receptor agonists, such as 8-OH-DPAT, reduce the firing rate of 5-HT afferents thereby reducing the release of 5-HT in terminal regions (Verge et al., 1985; Blier and de Montigny, 1987). Therefore, the suppression of nausea by CBD appears to be mediated by its reduction of LiCl-induced 5-HT in the IIC.

The LiCl-induced elevation of 5-HT in the IIC is the presumed trigger for the sensation of nausea. However, it is also possible that 5-HT is elevated in the IIC as a response to nausea induced behaviours displayed by the rat during the experience with LiCl. It is unlikely that the elevated 5-HT is the result of context-LiCl associations, given that the rats did not have any prior experience with LiCl in the context of the microdialysis chambers. As well, it is unlikely that the elevated 5-HT is due to the rats experiencing something aversive (not nausea per se), because (1) only drugs that produce emesis in other species produce conditioned gaping in rats (Parker 2014), (2) drugs which reduce 5-HT release [e.g., HT_1A_ agonists, CBD (Rock et al., 2012), and 8-OH-DPAT (Limebeer and Parker, 2003)] prevent LiCl-induced conditioned gaping in rats, (3) selective 5-HT lesions in the IIC prevent LiCl-induced conditioned gaping in rats (Tuerke et al., 2012a), (4) elevation of 2-AG in the IIC prevents LiCl-induced conditioned gaping in rats (Sticht et al., 2016) and reduces the elevated serotonin release in this region (data reported here). These all suggest that it is not an aversive event per se that produced the elevated 5-HT in the IIC, but the selective aversive event of a nauseating experience.

Although MJN110 reduces LiCl-induced conditioned gaping when delivered systemically (Parker et al., 2015) or intracranially into the IIC (Sticht et al., 2016), IIC administration of MJN110 did not prevent conditioned gaping produced by intracranial administration of the postsynaptic 5-HT_3_ receptor agonist, mCPBG. On the other hand, the postsynaptic 5-HT_3_ receptor antagonist, ondansetron administered to the IIC did prevent mCPG induced conditioned gaping as has been reported by Tuerke et al., 2012a). These results provide further support that the regulation of nausea by 2-AG in the IIC is via its action on presynaptic CB_1_ receptors which act to “turn off” the release of 5-HT from terminal endings. They further suggest that LiCl produces nausea by elevating 5-HT release in the IIC which in turn acts on postsynaptic 5-HT_3_ receptors in this region.

Not only did the unconditional nausea inducing agent, LiCl, produce elevation of 5-HT in the IIC, exposure to a LiCl-paired saccharin cue produced a conditional elevation of 5-HT in the IIC during the 20-min intraoral infusion while rats displayed the nausea-induced behavior of conditioned gaping. These results provide evidence that conditioned nausea is also produced by elevation of 5-HT in this region.

Although elevation of 2-AG in the IIC by MAGL inhibition reduced LiCl-induced conditioned gaping in rats, Sticht et al. (2016) found that fatty acid amide hydrolase (FAAH) inhibition in the IIC did not prevent LiCl-induced conditioned gaping. Indeed, FAAH inhibition in the IIC did not elevate AEA in this region, although it did elevate other fatty acids, oleoyl ethanolamide (OEA) and palmitoyl ethanolamide (PEA), which are also degraded by FAAH. These results suggest that the endogenous cannabinoid that regulates nausea within this brain region is 2-AG, not AEA. On the other hand, FAAH inhibition has been found to reduce LiCl-induced nausea when administered systemically (Rock et al., 2015; Parker et al., 2016). This effect may be mediated by a peripheral mechanism, since Rock et al. (2017) found that the peripherally restricted FAAH inhibitor, URB937, reduced LiCl-induced conditioned gaping, potentially by its action on the area postrema, an area of weakened blood brain barrier. Future research needs to address potential central sites of action of FAAH inhibition in the reduction of nausea.

Despite its prevalence, the treatment of nausea has lagged behind the treatment of vomiting. There is a pressing need to treat this distressing symptom. The results of these experiments provide a greater understanding of the neuroanatomical and neurochemical basis of nausea, and may be useful in identifying new treatments that act to reduce nausea-induced 5-HT release either by boosting the eCB system or by reducing IIC release of 5-HT.

## Synthesis

Reviewing Editor: Carmen Sandi, Swiss Federal Institute of Technology Decisions are customarily a result of the Reviewing Editor and the peer reviewers coming together and discussing their recommendations until a consensus is reached. When revisions are invited, a fact-based synthesis statement explaining their decision and outlining what is needed to prepare a revision will be listed below. Note: If this manuscript was transferred from JNeurosci and a decision was made to accept the manuscript without peer review, a brief statement to this effect will instead be what is listed below.

This solid, well-executed paper builds on previous work and knowledge to clarifying insula neurochemical mechanisms of nausea and conditioned disgust. It describes neurochemical signals in insula in nausea and conditioned taste disgust. Specifically, the authors test the hypothesis that elevation of 5-HT3 signals in visceral insula mediates nausea (such as triggered by systemic LiCl), whereas local 2-AG endocannabinoid signals suppress nausea. This could be prevented by systemic pharmacological disruption, which also blocked induction of aversive gapes to taste. Using microdialysis, they report that LiCl administration that serves as UCS to induce conditioned taste aversion elevates 5-HT in insula. Similarly, an aversive CS taste also elicited 5-HT elevations, after pairing with LiCl. Conversely they report that insula microinjection of 5HT3 antagonist ondansetron blocks LiCl ability to establish conditioned aversion to saccharin. Overall, the manuscript is scientifically sound and the conclusions supported by the evidence. It helps confirm the role of insula cortex in mediating nausea and conditioned disgust, and specifically pinpoint 5-HT mechanisms of nausea, and suppressive mechanisms that block nausea and disgust either via serotonin blockade or via cannabinoid stimulation. The authors have now successfully clarified previous points raised by the reviewers.
